# Long-term follow-up of a multimodal day clinic, group-based treatment program for patients with very high risk for complex posttraumatic stress disorder, and for patients with non-complex trauma-related disorders

**DOI:** 10.3389/fpsyt.2023.1152486

**Published:** 2023-06-15

**Authors:** Anke Bever-Philipps, Andrea Silbermann, Eva Morawa, Eva Schäflein, Mark Stemmler, Yesim Erim

**Affiliations:** ^1^Department of Psychosomatic Medicine and Psychotherapy, University Hospital of Erlangen, Friedrich-Alexander University Erlangen-Nürnberg (FAU), Erlangen, Germany; ^2^Institute of Psychology, Friedrich-Alexander University Erlangen-Nürnberg (FAU), Erlangen, Germany

**Keywords:** PTSD, CPTSD, follow-up, group therapy, day clinic, psychotherapy, trauma

## Abstract

**Objective:**

The present study examined the follow-up of a multimodal day clinic group-based therapy program for patients with trauma-related disorders and investigated potential differences for patients with classic PTSD versus cPTSD.

**Method:**

Sixty-six patients were contacted 6 and 12 months after discharge of our 8-week program and completed various questionnaires (Essen Trauma Inventory (ETI), Beck Depression Inventory-Revised (BDI-II), Screening scale of complex PTSD (SkPTBS), Patient Health Questionnaire (PHQ)-Somatization, as well as single items to therapy utilization and life events in the interim period). Due to organizational reasons a control group could not be included. Statistical analyses included repeated-measures ANOVA with cPTSD as between-subject factor.

**Results:**

The reduction of depressive symptoms at discharge was persistent at 6 and 12 months follow-up. Somatization symptoms were increased at discharge, but were leveled out at 6 months follow-up. The same effect was found for cPTSD symptoms in those patients with non-complex trauma-related disorders: Their increase of cPTSD symptoms was flattened at 6 months follow-up. Patients with a very high risk for cPTSD showed a strong linear reduction of cPTSD symptoms from admission to discharge and 6 months follow-up. cPTSD patients had a higher symptom load compared to patients without cPTSD on all time points and scales.

**Conclusion:**

Multimodal, day clinic trauma-focused treatment is associated with positive changes even after 6 and 12 months. Positive therapy outcomes (reduced depression, reduced cPTSD symptoms for patients with a very high risk for cPTSD) could be maintained. However, PTSD symptomatology was not significantly reduced. Increases in somatoform symptoms were leveled out and can therefore be regarded as side effects of treatment, which may be connected with actualization of trauma in the intensive psychotherapeutic treatment. Further analyses should be applied in larger samples and a control group.

## 1. Introduction

The effectiveness of psychotherapeutic treatment for Posttraumatic Stress Disorder (PTSD) can be considered proven (1, 2). Furthermore, group-based programs (3, 4) as well as inpatient treatments (5, 6) were shown to be effective in reducing PTSD symptomatology. For these treatments, follow-up examinations that address the question whether these effects can be maintained over a longer period of time are of important interest.

An increasing number of studies investigated long-term outcomes of treatments for patients with PTSD. Meta-analyses demonstrated long-term efficacy for adults (7) and youth (8). Kline and colleagues (7) did not find significant differences between treatment types from pretreatment to follow-up, but in the comparison of posttreatment to follow-up, exposure-based treatments (i.e., prolonged exposure, imaginal exposure) showed better results than other treatment types (cognitive behavioral therapies-mixed, cognitive processing therapy, cognitive therapy, EMDR). There was no link to trauma type, population type (i.e., military vs. civilian) or intended duration of treatment, suggesting efficacy for a wide variety of patients. Other studies showed encouraging results for long-term efficacy for various groups of patients, outpatient treatment types and different follow-up intervals (e.g., EMDR, cognitive processing therapy, cognitive-behavioral therapy, written exposure therapy) (9–16). One study also reported significant long-term improvements in depression, PTSD and anxiety symptoms 2.5 years after a mindfulness-based stress reduction (MBSR) program for adult survivors of childhood sexual abuse (17), a therapy component that was also included in our multimodal treatment program (see Section “Treatment description”). But, some studies had important limitations concerning the sample [e.g., exclusion of patients after childhood sexual abuse in König et al. (13)], which were overcome by the present examination.

Some studies demonstrated long-term efficacy for inpatient treatment programs for veterans or active duty military personnel with combat-related or non-combat-related PTSD (18–21). Outcome measures included not only PTSD scores or diagnostic status but also symptoms of depression, anxiety, problems with anger, alcohol difficulties and general functioning. Lampe and colleagues (22) examined an inpatient program for female survivors of childhood sexual abuse and demonstrated significant improvements compared to admission for global symptom load [measured by the Global Severity Index of the Symptom Checklist (SCL-90-R)], PTSD, depression and self-soothing ability, but dissociative symptoms remained unchanged.

For patients with very high symptom severity and impairment in everyday functioning, outpatient treatment often could not be sufficient, so that inpatient treatment is offered. In this group of patients, the ones with the diagnosis complex PTSD might play an important role.

The concept of complex PTSD was introduced by Herman (23) and describes a special symptom pattern which is caused by severe interpersonal trauma that often lasted for a long period, such as sexual abuse in childhood (23–25). cPTSD includes the classic PTSD symptoms intrusion, hyperarousal and avoidance, but goes beyond them and contains difficulties in emotion regulation (e.g., increased emotional reactivity, self-harming behavior), alterations in self-concept (e.g., disturbed feeling of identity, belief to live a shattered life or to be worthless, permanent feelings of guilt and shame), and relationship problems (e.g., inability to trust others or difficulties maintaining a stable relationship) (23, 24, 26). cPTSD will be included in ICD-11. Psychiatric burden and functioning impairment are often worse than in patients with classic PTSD (27). In the last years, a growing number of studies investigated the effectiveness of psychotherapeutic treatments for patients with cPTSD and demonstrated reductions in PTSD, depression, psychological distress, dissociation, and relationship problems (28–31). Other studies reported less treatment gain and lower recovery rates for patients with cPTSD compared to patients without cPTSD (25).

As the German healthcare system relies on diagnoses based on the ICD, we decided to follow this diagnostic system and to explore the potential influence of cPTSD on therapy outcome in follow-up.

Up to date, very few studies examined the long-term course of cPTSD patients after specialized treatment programs. We only found one study focusing explicitly on patients with cPTSD: Müller and Sachsse (32) investigated an inpatient treatment for women with cPTSD using integrative methods (psychodynamic imaginative trauma therapy, EMDR, elements of dialectical-behavioral therapy) and showed long-term improvements in disorder specific and adjoining symptoms as well as in coping behavior, quality of life and general psychological strain. Nevertheless, they stated that the “memory remains.” The study of Lampe and colleagues (22) included patients with type II trauma histories (98% emotional abuse in childhood, 100% emotional neglect, 65% physical abuse, 93% sexual abuse in childhood) and severe trauma-related symptomatology (90% borderline personality disorder, 85% PTSD, 44% dissociative identity disorder), so that this investigation further widens the encouraging long-term results for patients with complex forms of trauma-related disorders.

Beyond that, one has to keep in mind the above described difficulties of cPTSD patients concerning emotion regulation, relationships with others and self-esteem as well as their often higher symptom load, which could affect posttreatment as well as follow-up scores negatively. In a previous study, women with multiple abuse histories (combinations of physical, emotional and/or sexual abuse in childhood simultaneously or sequentially) and women who were blamed and rejected by their mothers for the childhood abuse were demonstrated to have lower long-term effects in depression, suicidality and self-esteem in a social work group therapy (16).

Overall, the data concerning long-term efficacy of treatments for cPTSD is only preliminary, up to date a clear statement cannot be made. Because of the high burden of impairment and stress which patients with cPTSD experience, the examination of long-term effects of specialized treatments is of great importance.

In comparison to inpatient treatment, day clinic treatment contains the same therapy intensity and multimodality, with the important difference that patients spend their therapy-free time at home. Therefore, day clinic treatment combines the advantages from outpatient and inpatient treatment, for example undergoing intensive multimodal and multiprofessional therapy but also staying connected to family and friends and to maintain safety and comfort by sleeping at home (a more detailed description of our program is given in Section “Treatment description”). However, day clinic treatment has seldom been investigated.

We only found a single study reporting long-term efficacy of a day clinic program: Drozdek and colleagues (33) examined the effects of a one-year phase-based trauma-focused multimodal multicomponent group therapy for PTSD in Iranian and Afghan asylum seekers and refugees in the Netherlands and found reductions of symptoms directly after treatment that continued up to 5 years posttreatment. After 5 years, all symptoms started to worsen, but remained lower than the baseline levels.

The present study reflects the long-term evaluation of our multimodal day clinic group-based treatment program for patients with trauma-related disorders. In a previous work (34), we showed that after 8 weeks of treatment, depressive symptoms were reduced and post-traumatic growth was increased. Additionally, we examined potential differences in outcome for patients with versus without cPTSD: For patients with cPTSD, depressive as well as cPTSD symptoms were significantly reduced, perceived social support was increased. Contrary to our expectations, somatoform symptoms were increased after therapy, as well as cPTSD symptoms for those patients without cPTSD.

The aim of the present study was to evaluate the long-term course of symptomatology in 66 patients with complex versus non-complex trauma-related disorders who underwent a multimodal day clinic group-based treatment. As such, it is one of the first studies in this field.

We hypothesized a maintenance or further reduction of symptom scores (PTSD, cPTSD, depressive and somatoform symptoms). Additionally, we explored potential differences in the follow-up courses for patients with versus without very high risk for complex PTSD. As a research question, we examined whether patients with a very high risk for cPTSD show trajectories with higher symptom scores than patients with non-complex trauma-related disorders.

## 2. Materials and methods

### 2.1. Treatment description

An exact description of our multimodal treatment can be found in our previous article (34). The study was conducted in a psychosomatic day clinic of a German University Hospital. Exclusion criteria were acute psychosis, acute suicidality, present substance abuse or dependence, clear underweight (BMI < 17 kg/m^2^), unstable social conditions such as homelessness, a journey to the day clinic of more than 1 h, contact to the offender, or not being able to participate in groups (e.g., extreme dissociation). The goal was to treat patients with trauma-related disorders (PTSD, cPTSD, and anxiety, affective, somatoform, or personality disorders relating to traumatic experiences in the past). Patients were treated in a closed group format with trauma-focused integrative therapy composed of individual and group psychotherapy with cognitive-behavioral and psychodynamic approaches, trauma-specific psychoeducation, skills training, mindfulness and relaxation methods, art therapy, concentrative movement therapy and pharmacological therapy if needed. The amount of stabilization and confrontational methods was chosen by the therapists depending on patients’ individual therapy goals and condition, but the minimum weekly dose of psychotherapy was set according to German health insurances regulations and fulfilled the intensive coordinated psychotherapy. Each group consisting of 7 patients stayed together for the 8 weeks of treatment. Therapy took place from Monday to Friday from 9 a.m. to 4 p.m. As such, patients underwent an intensive psychotherapeutic program but could also stay connected with their families and friends and their home, which reflected an important safety anchor during the strenuous treatment. Furthermore, day clinic treatment facilitates transfer from therapy to everyday life because therapy content can immediately be practiced in patients’ interactions at home. Day clinic treatment therefore combines the advantages of inpatient and outpatient treatment.

### 2.2. Design and procedure

The study was conducted in a longitudinal, naturalistic design (i.e., observational study). Every patient fulfilling inclusion criteria and therefore being admitted to the trauma-focused treatment in our day clinic was asked to participate in the study. At approval, written informed consent was obtained from every participant. Seventy-three patients were admitted and asked to participate, 66 (90.4%) gave their written consent. The study was approved by the ethics committee of the Medical Faculty of the Friedrich-Alexander University Erlangen-Nürnberg (FAU) (153_18B).

**Figure 1:**
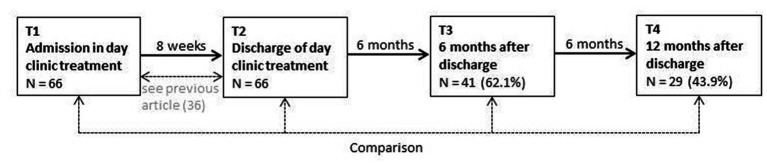
Study design.

Figure 1 shows the study design with all four time points and the amount of participants.

At T1 and T2, patients filled out the questionnaires, which were also used for individual diagnostics and therapy planning, in the day clinic. Before T3 and T4, patients were contacted on telephone by the first author. A standardized protocol for telephone conversation was maintained. Patients were reminded of the study format and its goals and were asked to fill out and send back the questionnaires in the provided franked envelope. Furthermore, it was highlighted that patients could ask for supportive psychotherapeutic counseling if required through answering the questionnaires.

### 2.3. Instruments

The Essen Trauma Inventory [ETI; (35, 36)] includes a list of 15 potentially traumatic experiences, questions concerning objective and subjective threat to life (criteria A1 and A2), and 23 questions about symptoms on the subscales intrusion, hyperarousal, avoidance, and dissociation. Symptoms are rated on a 4-point Likert-type scale ranging from “0 = never” to “3 = very often.” Clinically relevant PTSD is indicated by existence of a traumatic experience, fulfilled A1 and A2 criteria as well as a symptom sum score ≥ 27 when adding the items of the subscales intrusion, hyperarousal, and avoidance. Tagay and colleagues (35) confirm the ETI to be reliable and valid. Objectivity, reliability and validity were also confirmed by a study examining German soldiers (37). For our analyses, we used the total sum score including all four subscales. Cronbach’s alpha was α = 0.95 for T1, α = 0.96 for T2, α = 0.97 for T3, and α = 0.97 for T4.

The Screening of complex PTSD [in German, SkPTBS, (38)] captures potentially traumatic experiences, risk and protective factors like frequency and duration, type of causation (e.g., family member, accident) and complex PTSD symptoms which are composed of difficulties in affect and impulse control (e.g., self-calming ability, anger control), interactional problems (e.g., ability to trust another person), negative self-image (e.g., feelings of guilt, belief to live a shattered life), and dissociative symptoms. Symptoms are rated on a 7-point Likert scale ranging from “0 = does not apply at all” to “6 = totally fits.” The authors of the SkPTBS offer a matrix for analysis and comparative values as well as a division into very high, high and low risk for complex PTSD. The SkPTBS was demonstrated to be reliable, one-dimensional, and valid (38). The factor structure as well as the good psychometric properties were replicated (39). In our study, Cronbach’s alpha of the symptom scale was α = 0.89 for T1, α = 0.90 for T2, α = 0.92 for T3, and α = 0.91 for T4.

The Patient Health Questionnaire: somatization module [PHQ-15, (40)] contains 13 items measuring somatic symptoms with the response options “not bothered at all” (0), “bothered a little” (1), or “bothered a lot” (2). The sum score additionally includes two items from the depression module which assess sleeping disorders and tiredness. Sum scores of 5, 10, and 15 represent cut-offs for mild, moderate, and severe levels of somatization, with a possible range of 0 to 30 points for the sum score. The PHQ-15 was shown to have good psychometric properties (41–43). Concerning the factor structure, a general factor was found in different samples (44) and was replicated (45). In our study, Cronbach’s alpha was α = 0.79 for T1, α = 0.84 for T2, α = 0.88 for T3, and α = 0.89 for T4.

The Beck Depression Inventory-Revised [BDI-II; (46); German version (47)] contains 21 items measuring depressive symptoms such as sadness, feelings of guilt, insufficiency or worthlessness, and reduced interest in others as experienced within the last 2 weeks. Symptoms are rated on a 4-point scale ranging from 0 to 3 anchored with example sentences. Sum scores reflect no (0 to 8), minimal (9 to 13), mild (14 to 19), moderate (20 to 28), and severe (29 to 63) depression, respectively. The BDI-II was reported to be reliable and valid (48, 49). In our study, Cronbach’s alpha was α = 0.90 for T1, α = 0.93 for T2, α = 0.95 for T3, and α = 0.96 for T4.

### 2.4. Statistical analysis

All analyses were conducted with SPSS Statistics 26 (IBM Statistics, Chicago, IL, USA). We computed frequencies to show descriptive statistics. Classification of patients as having a very high risk for cPTSD was performed as indicated by the authors of the SkPTBS (see instruments (38); i.e. reaching a score of 28.19 or higher). Follow-up evaluation was conducted using repeated-measures ANOVA with Bonferroni-adjusted post-hoc analysis, with cPTSD as between-subject factor in later analyses. In the analyses examining cPTSD as between-subject factor, we only used T1 to T3 because of the small number of T4 responders and the further division in groups for the analyses. A replacing of missing values did not seem appropriate to us, as the analysis of the pattern showed that data were not missing at random, but that there was a systematic loss of data at T4. Furthermore, replacing values with the last value carried forward method would have positively biased the analyses, as a maintaining of decreased values after discharge would confirm our hypothesis of therapy success.

Significance level in all analyses was *p* ≤ 0.05, except for the case of alpha error correction.

### 2.5. Dropout analysis

We used *t*-tests to compare T3 and T4 respondents, respectively, with patients who did not send back follow-up questionnaires concerning their ETI, SkPTBS, BDI-II and PHQ values at T1 and T2. For T3, we did not find significant differences, except for scores of PHQ Somatization. T3 respondents had significantly lower scores at T2 compared to non-respondents (*t* = 2.21, *p* = 0.03). For T4, analyses showed that T4 respondents had significantly lower BDI-II scores at T2 than non-respondents (*t* = 2.25, *p* = 0.03).

### 2.6. Sample characteristics

Sixty-six patients participated in the multimodal day clinic treatment program (*n* = 55 female patients). Of those, 41 responded to our follow-up questionnaires at T3 (62.1%, *n* = 36 female patients). At T4, 29 patients participated (43.9%, *n* = 25 female patients). Sixteen participants of T3 (40.0%) were classified as having a very high risk of cPTSD. At T4, the number of patients with very high risk for cPTSD was 11 (39.3%).

The following diagnoses were given during the treatment program: PTSD (*n* = 53; 22 of them with the appendix “complex”), “other reactions to severe stress” (*n* = 7), personality disorders (*n* = 11), depressive disorders (*n* = 65), obsessive compulsive disorder (*n* = 6), somatoform disorders (*n* = 15), anxiety disorders (*n* = 11), eating disorders (*n* = 4), substance abuse (*n* = 3), dissociative disorders (*n* = 2), impulse control disorders (*n* = 1) and hyperkinetic disorders (*n* = 1). Comorbid diagnoses were predominant.

Information about sociodemographic factors and pre-treatment factors can be found in our previous article (34).

Table 1 reveals post-treatment therapy utilization and life changes. Most patients received outpatient psychotherapy, but only a low amount was treated with exposure-based methods. Inpatient admissions were rare. Many patients experienced stressful or critical life-events in-between, but only in few cases these included further traumatic events.

**Table 1:** Post-treatment variables.

	T3 (*N* = 41)		T4 (*N* = 29)	
	*N*	%	*N*	%
Critical life events in-between^1^	25	64.1	18	66.7
Outpatient treatment in-between	34	82.9	26	89.7
Outpatient trauma exposition treatment in-between	9	23.1	6	20.7
Inpatient treatment in-between (including repeated admission in our day clinic^2^)	5	12.5	3	10.3
Change in medication	16	39.0	8	27.6

^1^These include stressful events as well as traumatic events in few cases. ^2^This only applies for 2 (T3) and 1 (T4) patients, respectively, so that these were left in the sample.

## 3. Results

### 3.1. Follow-up evaluation

Table 2 shows the results of the follow-up evaluation over all 4 time points for ETI, BDI-II, SkPTBS, and PHQ Somatization. Graphic illustrations for each symptom category can be found in Supplementary material.

**Table 2:** Follow-up evaluation (all patients).

	*N*	*F* (df)	*p*	*d*
ETI Total Score	27	2.83 (2.02, 52.50)	0.068	0.33
BDI-II	29	6.01 (3, 84)	0.001	0.46
SkPTBS	28	2.22 (3, 81)	0.092	0.29
PHQ Somatization	28	1.81 (3, 81)	0.152	0.26

The table presents the number of patients included in the analysis, *F*-values and degrees of freedom, *p*-values and effect sizes (Cohen’s d).

PTSD symptoms did not change significantly over time (see Table 2). All pairwise comparisons between the time points were nonsignificant. The effect sizes for the pairwise comparisons were as follows, showing small to medium positive effects in symptom reduction when comparing T1 and T2 with T3 and T4: T1-T2 *d* < 0.01, T1-T3 *d* = −0.28, T1-T4 *d* = −0.29, T2-T3 *d* = −0.29, T2-T4 *d* = −0.29, T3-T4 *d* = −0.01.

For depressive symptoms, we found significant changes over time (see Table 2). Pairwise comparisons showed significant differences between T1 and every other time point (T2: *p* = 0.002, T3: *p* = 0.013, T4: *p* = 0.015) with the following effect sizes: T1-T2 *d* = −0.51, T1-T3 *d* = −0.49, T1-T4 *d* = −0.55, T2-T3 *d* < 0.01, T2-T4 *d* = −0.07, T3-T4 *d* = −0.07.

cPTSD symptoms did not change significantly (see Table 2). Pairwise comparisons were nonsignificant (T1-T2 *d* = −0.06, T1-T3 *d* = −0.49, T1-T4 *d* = −0.19, T2-T3 *d* = −0.42, T2-T4 *d* = −0.13, T3-T4 *d* = 0.27).

For somatization symptoms, there was no significant change over time (see Table 2). Furthermore, pairwise comparisons were nonsignificant, but showing medium effect sizes in different directions (T1-T2 *d* = 0.26, T1-T3 *d* = −0.21, T1-T4 *d* = −0.11, T2-T3 *d* = −0.44, T2-T4 *d* = −0.34, T3-T4 *d* = 0.09).

### 3.2. CPTSD as influence factor in follow-up

For the following analyses, we used T1 to T3 because of the smaller number of respondents at T4, which prevented a further division into subgroups.

**Figure 2:**
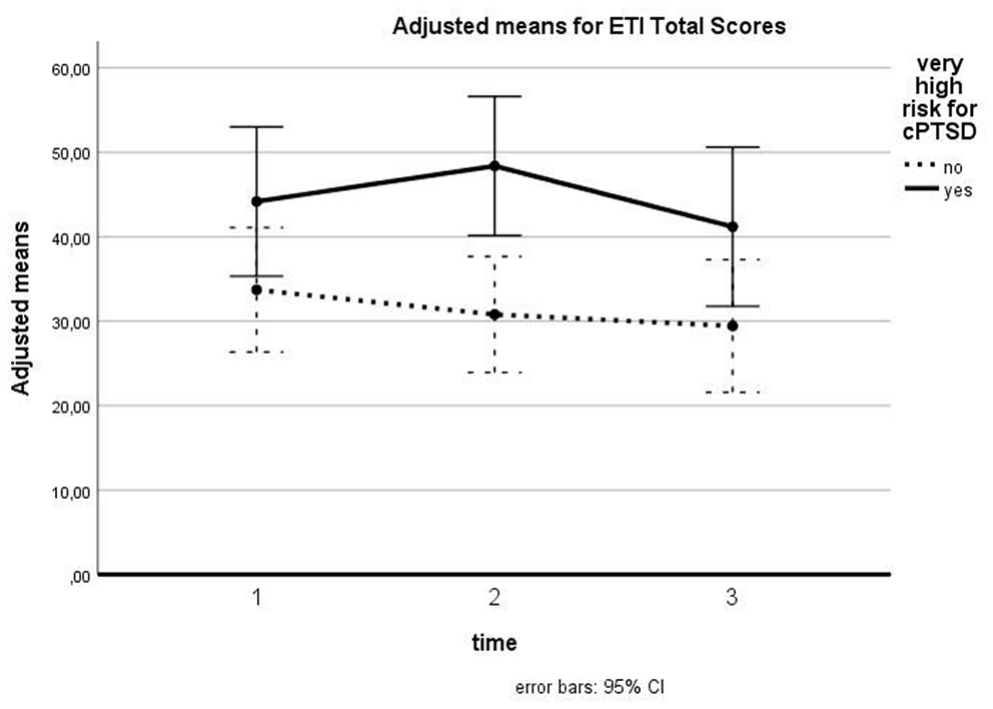
Influence of cPTSD on follow-up for ETI total scores. The illustration presents adjusted means of ETI total scores with error bars (95% confidence interval) for patients with versus without very high risk for cPTSD for T1, T2, and T3.

For PTSD symptoms, we found a nonsignificant time effect [*F* (1.57, 58.18) = 2.303, *p* = 0.120, *d* = 0.25] as well as a nonsignificant interaction between time and cPTSD [*F* (1.57, 58.18) = 1.559, *p* = 0.221, *d* = 0.20]. Pairwise comparisons between time points were also nonsignificant. The between-subject effect of cPTSD was shown to be significant [*F* (1, 37) = 6.730, *p* = 0.014, *d* = 0.43], demonstrating higher PTSD symptomatology for patients with very high risk for cPTSD (see Figure 2).

**Figure 3:**
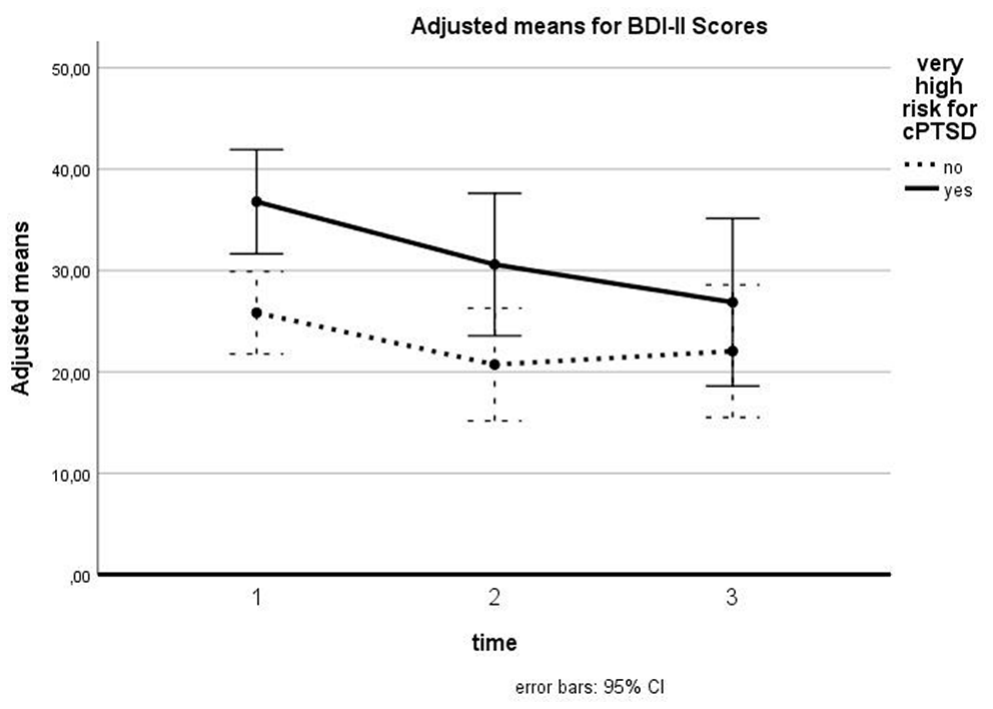
Influence of cPTSD on follow-up for BDI-II scores. The illustration presents adjusted means of BDI-II scores with error bars (95% confidence interval) for patients with versus without very high risk for cPTSD for T1, T2, and T3.

Depression symptoms were shown to be reduced over time for all patients [*F* (2, 74) = 9.189, *p* < 0.001, *d* = 0.50], with significant pairwise comparisons between T1 and T2 (*p* = 0.003) and T1 and T3 (*p* = 0.003). The difference between T2 and T3 was nonsignificant. There was no significant interaction between time and cPTSD [*F* (2, 74) = 1.834, *p* = 0.167, *d* = 0.22]. The between subject effect for cPTSD was significant [*F* (1, 37) = 4.825, *p* = 0.034, *d* = 0.36], showing higher scores for patients with cPTSD (see Figure 3).

**Figure 4:**
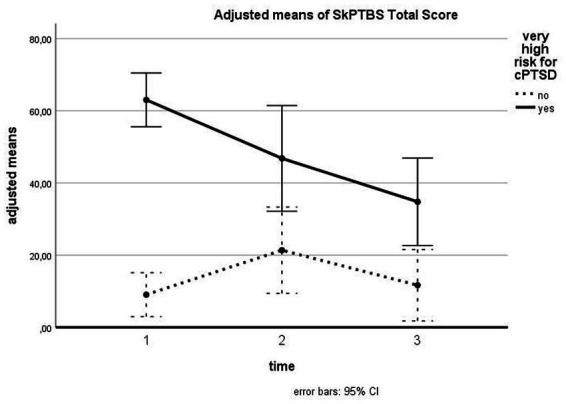
Influence of cPTSD on follow-up of SkPTBS total scores. The illustration presents adjusted means of SkPTBS total scores with error bars (95% confidence interval) for patients with versus without very high risk for cPTSD for T1, T2, and T3.

Concerning cPTSD symptoms, we found a significant time effect [*F* (2, 76) = 5.068, *p* = 0.009, *d* = 0.37]. Pairwise comparisons indicated a significant difference between T1 and T3 (*p* = 0.008), the differences between T1 and T2 and T2 and T3 were nonsignificant. We found a significant interaction between time and cPTSD [*F* (2, 76) = 7.850, *p* = 0.001, *d* = 0.45] as well as a significant between-subject effect for cPTSD [*F* (1, 38) = 37.196, *p* < 0.001, *d* = 0.99]. Figure 4 shows the course of cPTSD symptomatology for patients with and without cPTSD: while patients without very high risk for cPTSD showed a slight increase from T1 to T2 which then disappeared reaching the baseline level at T3, patients with a very high risk for cPTSD demonstrated a linear reduction of cPTSD symptoms above all time points. Pairwise comparisons showed that for patients with a very high risk for cPTSD, the differences between T1 and T2 (*p* = 0.051) and between T1 and T3 (*p* = 0.008) were significant.

**Figure 5:**
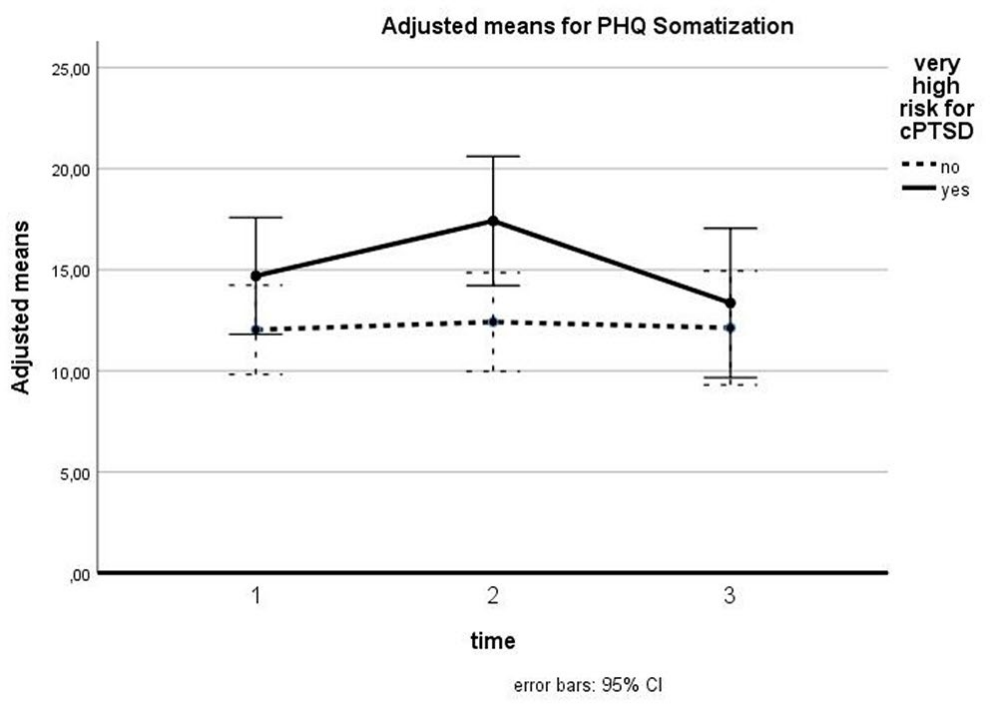
Influence of cPTSD on follow-up for PHQ Somatization. The illustration presents adjusted means of PHQ somatization scores with error bars (95% confidence interval) for patients with versus without very high risk for cPTSD for T1, T2, and T3.

For somatization symptoms, we found a significant time effect for all patients [*F* (1.69, 60.85) = 4.091, *p* = 0.027, *d* = 0.34] with significant differences between T1 and T2 (*p* = 0.047) and T2 and T3 (*p* = 0.029). The difference between T1 and T3 was nonsignificant. The interaction between time and cPTSD was nonsignificant [*F* (1.69, 60.85) = 2.946, *p* = 0.069, *d* = 0.29]. There was no significant between-subject effect for cPTSD [*F* (1, 36) = 2.649, *p* = 0.112, *d* = 0.27]. Figure 5 shows the course of somatization symptoms for patients with and without cPTSD. For patients with a very high risk for cPTSD, a slight increase from T1 to T2 can be observed, which is leveled out at T3.

## 4. Discussion

### 4.1. Main results

The aim of the present study was to examine the courses of various symptoms 6 and 12 months after multimodal, day clinic treatment. The reduction of depressive symptoms between T1 and T2 was persistent over 12 months after treatment. PTSD and cPTSD symptoms did not change significantly. Somatization symptoms showed significant changes over time: The increase between T1 and T2 was leveled out at T3, resulting in scores similarly to those at T1. Additionally, we explored potential differences in the course of patients with versus without very high risk for cPTSD. PTSD and depressive symptoms were higher for patients with a very high risk for cPTSD. cPTSD symptoms were reduced over time for cPTSD patients. For patients with noncomplex PTSD, the increase in cPTSD symptoms between T1 and T2 was reduced to T3, resulting in the low level observed at T1.

### 4.2. Nonsignificant changes in PTSD symptoms

PTSD symptoms remained on a relatively stable level throughout the examined time points, showing only a trend to change significantly (*p* = 0.068 in the analysis with all patients) with slight reductions in the graphic illustration. This result is contrary to our expectations as well as to many previous studies that reported reductions of PTSD symptomatology (7, 22, 33). One reason for the nonsignificant course could be that our study sample was too small to detect significant changes, resulting only in a statistical trend, but a moderate effect size. The effect sizes of the pairwise comparisons indicated medium positive effects when comparing T1 and T2 with T3 and T4. Furthermore, our program did not explicitly include confrontational methods for every patient: The use of confrontational methods was individually planned concerning to the therapy goals and condition of patients. In a meta-analytic study, exposure-based treatments showed better results than other treatment types when comparing posttreatment to follow-up (7). Consequently, it is possible that our treatment did not contain enough confrontational methods to reduce PTSD symptoms significantly. Our examination showed that although many patients received outpatient psychotherapy, only a very low amount received a specialized trauma-oriented treatment after discharge of our program (see Table 1). But, this would reflect the recommended first-line treatment for PTSD (50). Therefore, we installed a phase-based interval treatment in the meantime, with one day clinic program for stabilization and improvement of overall functioning and one program for specialized confrontational therapy. An examination of this concept is still outstanding.

### 4.3. Maintenance of the reduction of depression symptoms

The reduction of depressive symptoms at the end of treatment was maintained during follow-up, with medium effect sizes both in analyses with 6 months and in those with 12 months. Patients were able to prolong the therapy success into their everyday life. These results resemble previous findings from the literature (19, 22) and reflect important improvements in patients’ quality of life and overall functioning, as nearly all patients in our study were diagnosed with depression.

### 4.4. Side effects during treatment

The unexpected increase of somatization symptoms at discharge was vanished at T3 and T4, respectively. Hence, this increase can be regarded as reflecting temporary side effects during treatment. Schäflein et al. (51, 52) have shown that highly-dissociative PTSD patients tend to tune out bodily, e.g., interoceptive and psychophysiological signals. Thus, the temporary increase in bodily misperceptions might be interpreted as an increase in self-perception, which might be especially intense directly after trauma-oriented psychotherapy. Later at follow-up, these perceptions might be more integrated and thus less intrusive. In our dense therapy program, patients were confronted with their problems and traumatic events as well as with the descriptions of other participants, which could have been very strenuous and agitating. This could have resulted in more physical complaints like feeling exhausted or feeling pain. It seems that after treatment, when therapy effects settle and stabilize in patients’ everyday life, these side effects disappear. Another important factor is the neglect of the body over the years and the approach of psychotherapists and medical doctors to the consequences of neglect. In our study, there were many female patients who had not taken advantage of preventive examinations at the dentist and the gynecologist, which are free of charge in Germany. They were encouraged to do so during the day-clinic therapy. Afterwards they became more concerned with these bodily aspects. It remains to be investigated whether the complex form of post-traumatic stress disorder, which by definition lasts longer, is accompanied by more biological changes, e.g., in the hypothalamo-pituitary axis, concerning cortisol and interleukins. This could mean that in the group of the complex traumatized patients more changes of the cortisol axis as well as of the cytokines have taken place and these lead to more somatoform complaints, e.g., to pain and sleep disturbances. In an own study, we found that a higher response to standardized stress stimuli (Trier Stress Test) in the form of higher secretion of interleukin-6 was associated with a worse outcome in terms of depressiveness (BDI measured). These biological and somatic components may play a special role in complex PTSD patients (53). Another possible explanation is that the increase of somatization symptoms between T1 and T2 could be a bias in patients’ answers due to the intensive attention on and consideration of patients’ difficulties and condition during treatment. After discharge, when patients reconnect with their everyday life, this focus might again be reduced.

This could also explain the unexpected increase of cPTSD symptoms from T1 and T2 for patients with non-complex trauma-related disorders: Their high ratings on the SkPTBS at discharge could reflect a bias due to their occupation with this diagnosis and their co-participants in the groups. This effect is leveled out after 6 months, resulting in the same low level as at admission.

### 4.5. Follow-up for patients with a very high risk for cPTSD

The analyses showed that patients with a very high risk for cPTSD had a higher PTSD and depressive symptom load, resembling results in previous literature (27). Nevertheless, cPTSD patients could also maintain their success in reducing depressive symptoms. Somatization symptoms were also leveled out. For cPTSD symptoms, we found a strong effect with a graphically linear reduction from T1 to T2 and T3. This demonstrated that emotion regulation difficulties, problems with other people and low self-worth could be addressed through our program, which may have led to improvements for cPTSD patients’ quality of life and functioning. The positive effects of our multimodal group program can be maintained and extended after treatment, which reflects important changes for patients confronted with this severe trauma-related disorder. One explanation for this decrease of cPTSD symptoms can be found in the group-based form of our treatment: By spending their time and overcoming difficulties together with their co-patients, they might have learned social skills and might have gotten more in touch with other people, which could have heightened feelings of affiliation and being accepted, which then would positively influence one’s self-worth. Additionally, emotion regulation and social competencies were intensively trained in our program, which could also have strengthened these skills in our patients. Lastly, self-soothing abilities and trust in one’s self-efficacy could have been promoted by program modules like mindfulness, relaxation methods or body psychotherapy.

### 4.6. Limitations and strengths

The limitations of our study are: The relatively low number of participants did not allow us to compute further analyses with influencing factors like outpatient treatment or changes in medication during follow-up. Furthermore, we were not able to include a control group due to organizational reasons. Symptoms were assessed with self-rating instruments and not with structured interviews, which could have resulted in a potential bias in the analyses.

Despite these limitations, our study has various strengths: It is one of the first to examine a multimodal day clinic treatment like it is conducted in many German hospitals. Its naturalistic design allows a high external validity and generalizability, demonstrating how treatments work in the field. The inclusion of cPTSD as an influence factor in our analyses is also a strength of the study, as cPTSD patients have specific difficulties, i.e., in interaction with other people, which influence treatment and outcome. The present study therefore expands previous research and adds to the understanding of clinical practice and methods.

Future research should include a higher number of participants, a control group and structured interviews to measure symptomatology.

### 4.7. Conclusion

This study showed that multimodal, day clinic trauma-focused treatment was associated with positive changes over 6 and 12 months after treatment, respectively. Positive therapy outcomes (reduced depression, reduced cPTSD symptoms for patients with a very high risk for cPTSD) could be maintained. As the treatment of patients with cPTSD becomes more and more important, the finding of decreased cPTSD symptoms for patients with a very high risk for cPTSD is highly relevant. In the follow-up of our treatment, increases in somatoform symptomatology were leveled out and can therefore be regarded as side effects of treatment, which were connected with actualization of trauma in the intensive psychotherapeutic treatment. PTSD symptoms did not change significantly. An investigation of our newly adapted interval program with more confrontational methods is planned.

## Data availability statement

The raw data supporting the conclusions of this article will be made available by the authors, without undue reservation.

## Ethics statement

The studies involving human participants were reviewed and approved by ethics committee of the Medical Faculty of the Friedrich-Alexander University Erlangen-Nürnberg (FAU) (153_18B). The patients/participants provided their written informed consent to participate in this study.

## Author contributions

AB-P, AS, and YE conceived and designed. AB-P conducted, analyzed, and wrote this manuscript. YE, AS, EM, ES, and MS provided feedback and mentorship on each stage of the research design and implementation, including a full review, and provision of feedback on the final manuscript. All authors contributed to the article and approved the submitted version.

## Conflict of interest

The authors declare that the research was conducted in the absence of any commercial or financial relationships that could be construed as a potential conflict of interest.

## Publisher’s note

All claims expressed in this article are solely those of the authors and do not necessarily represent those of their affiliated organizations, or those of the publisher, the editors and the reviewers. Any product that may be evaluated in this article, or claim that may be made by its manufacturer, is not guaranteed or endorsed by the publisher.
