# Supplementary material for: Long-term follow-up of a multimodal day clinic, group-based treatment program for patients with very high risk for complex posttraumatic stress disorder, and for patients with non-complex trauma-related disorders
Source: Front Psychiatry. 2023 Jun 15;14:1152486. doi: 10.3389/fpsyt.2023.1152486 (PMC10311064; doi:10.3389/fpsyt.2023.1152486)

Supplementary Material

Figure 6. Follow-Up Evaluation of ETI Total Scores.

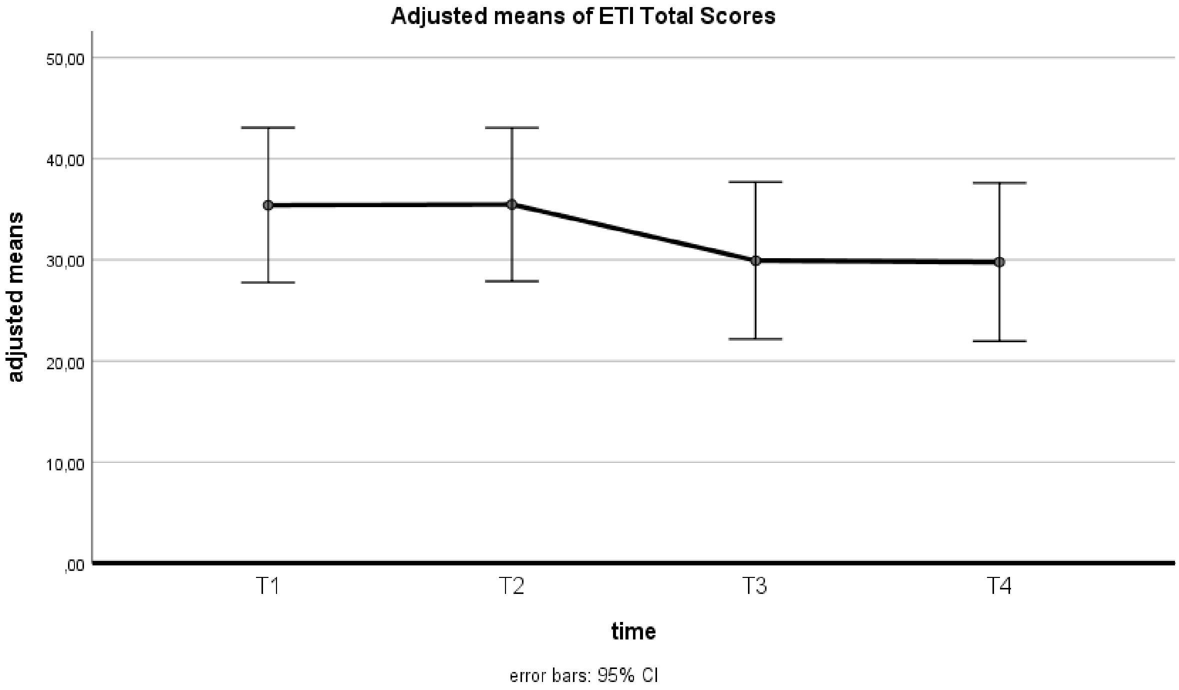

Figure 7. Follow-Up Evaluation of BDI-II Scores.

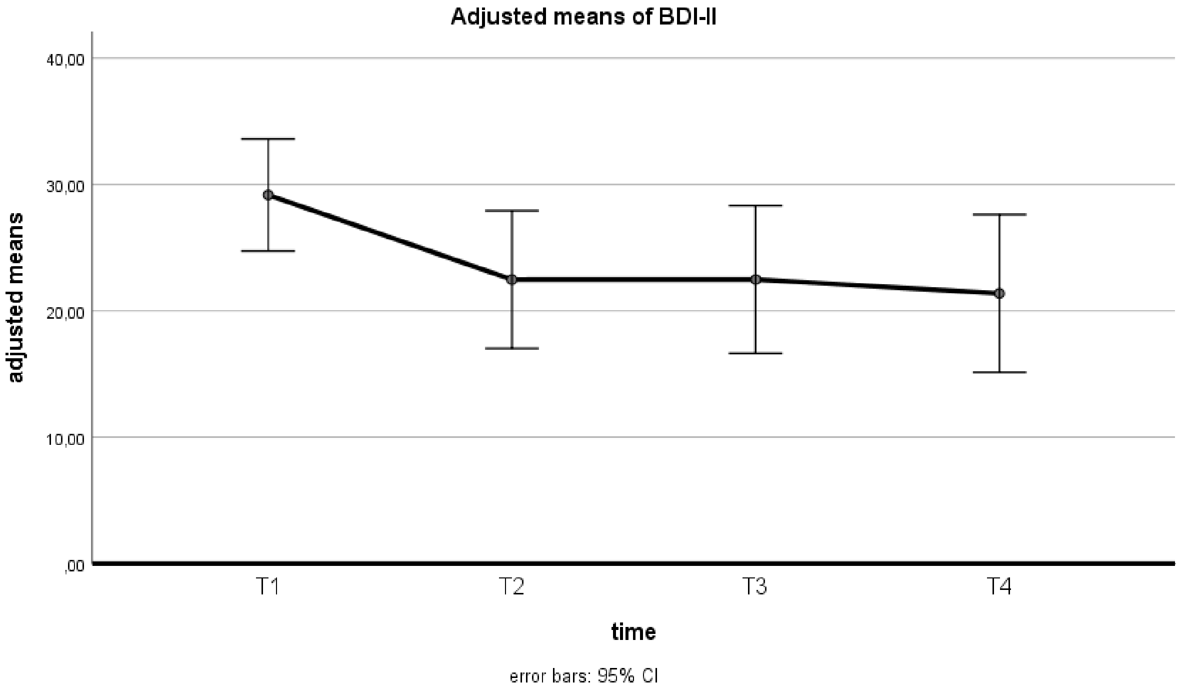

Figure 8. Follow-Up Evaluation of SkPTBS Total Scores.

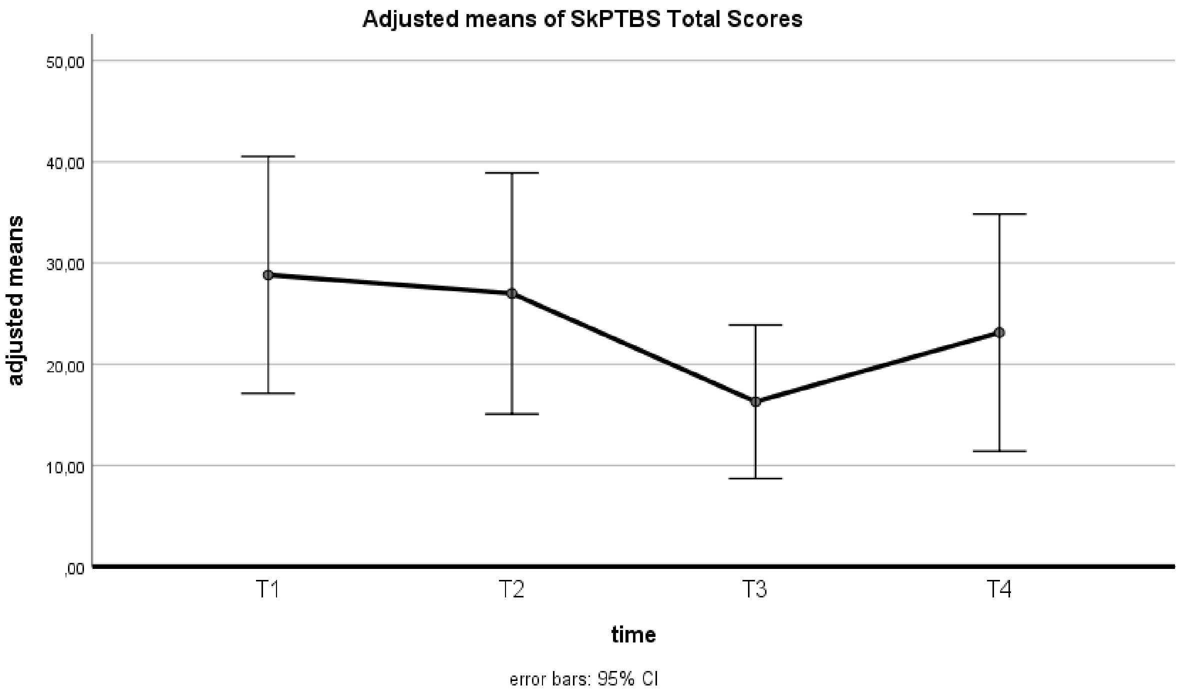

Figure 9. Follow-Up Evaluation of PHQ-Somatization.

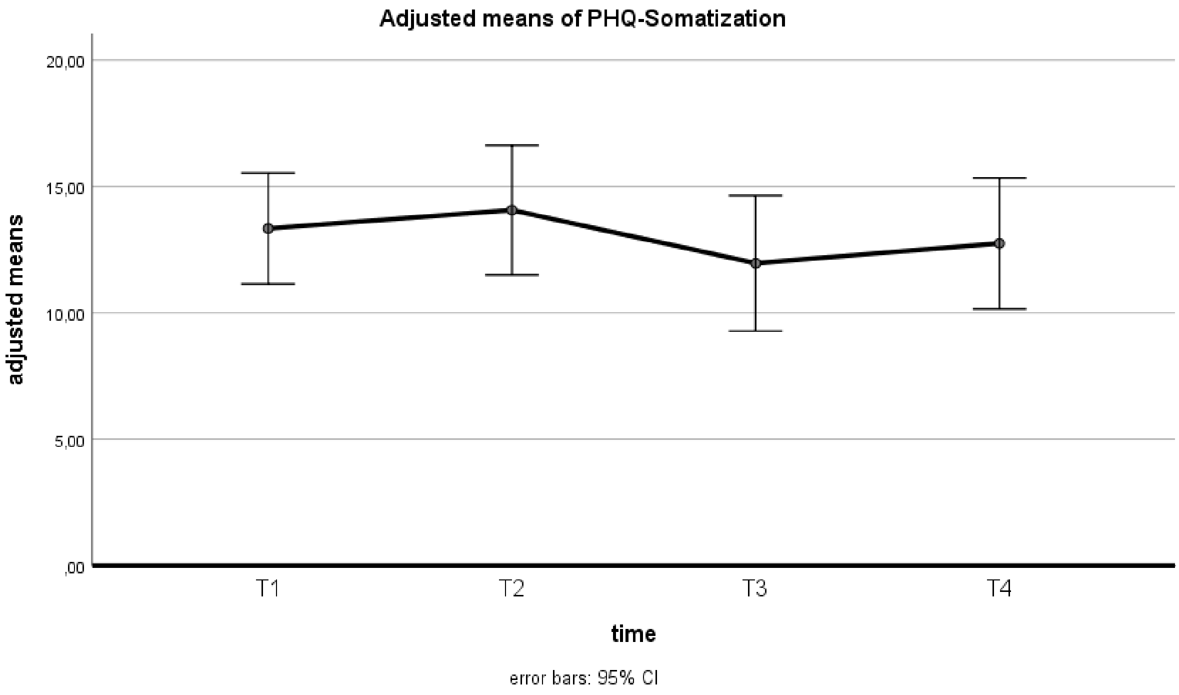

Supplement: Supplementary file 1 [file Data_Sheet_1.pdf]
